# Survival of a surrogate African swine fever virus-like algal virus in feed matrices using a 23-day commercial United States truck transport model

**DOI:** 10.3389/fmicb.2022.1059118

**Published:** 2022-12-09

**Authors:** Amanda Palowski, Cecilia Balestreri, Pedro E. Urriola, Jennifer L. G. van de Ligt, Fernando Sampedro, Scott Dee, Apoorva Shah, Haile F. Yancy, Gerald C. Shurson, Declan C. Schroeder

**Affiliations:** ^1^Department of Veterinary Population Medicine, College of Veterinary Medicine, University of Minnesota, St. Paul, MN, United States; ^2^Department of Animal Science, College of Food Agricultural and Natural Resource Sciences, University of Minnesota, St. Paul, MN, United States; ^3^Environmental Health Sciences Division, School of Public Health, University of Minnesota, Minneapolis, MN, United States; ^4^Pipestone Applied Research, Pipestone Veterinary Services, Pipestone, MN, United States; ^5^SAM Nutrition, Eden Prairie, MN, United States; ^6^U.S. Food and Drug Administration, Center for Veterinary Medicine, Office of Research, Laurel, MD, United States; ^7^School of Biological Sciences, University of Reading, Reading, United Kingdom

**Keywords:** African swine fever virus, *Emiliania huxleyi* virus, NCLDVs, feed, transport, viability PCR

## Abstract

African swine fever virus (ASFV) is a member of the nucleocytoplasmic large DNA viruses (NCLDVs) and is stable in a variety of environments, including animal feed ingredients as shown in previous laboratory experiments and simulations. *Emiliania huxleyi* virus (EhV) is another member of the NCLDVs, which has a restricted host range limited to a species of marine algae called *Emiliania huxleyi*. This algal NCLDV has many similar morphological and physical characteristics to ASFV thereby making it a safe surrogate, with results that are applicable to ASFV and suitable for use in real-world experiments. Here we inoculated conventional soybean meal (SBMC), organic soybean meal (SBMO), and swine complete feed (*CF*) matrices with EhV strain 86 (EhV-86) at a concentration of 6.6 × 10^7^ virus g^−1^, and then transported these samples in the trailer of a commercial transport vehicle for 23 days across 10,183 km covering 29 states in various regions of the United States. Upon return, samples were evaluated for virus presence and viability using a previously validated viability qPCR (V-qPCR) method. Results showed that EhV-86 was detected in all matrices and no degradation in EhV-86 viability was observed after the 23-day transportation event. Additionally, sampling sensitivity (we recorded unexpected increases, as high as 49% in one matrix, when virus was recovered at the end of the sampling period) rather than virus degradation best explains the variation of virus quantity observed after the 23-day transport simulation. These results demonstrate for the first time that ASFV-like NCLDVs can retain viability in swine feed matrices during long-term transport across the continental United States.

## Introduction

Foreign animal diseases, such as African swine fever virus (ASFV), pose a significant threat to the United States pork industry because contaminated feed ingredients, pork products, and humans can all be potential sources of disease introduction (Dee et al., 2014; Álvarez et al., 2019; Niederwerder et al., 2019; Dee et al., 2020a). Some RNA viruses such as Seneca virus A (SVA), Porcine reproductive and respiratory syndrome virus (PRRSV), and Porcine epidemic diarrhea virus (PEDV), have been shown to survive in feed ingredients and infect pigs under experimental conditions (Dee et al., 2014; Niederwerder et al., 2019; Dee et al., 2020b). Laboratory-based studies using experimentally inoculated ASFV in conventional and organic soybean meal, choline chloride, and complete feed have shown that the virus can survive for extended periods of time under simulated transoceanic shipping model conditions (Dee et al., 2018; Stoian et al., 2019; Dee et al., 2022). However, because ASFV is a highly contagious virus (Penrith, 2009; Costard et al., 2013; Oura, 2019), and countries such as those in the Americas, Australia and New Zealand where the disease is still absent,^1^ research with ASFV can only be conducted in a highly restricted biosecurity level 3 facility. Consequently, this has resulted in only a few laboratories in the world that have regulatory approval to work with this virus (Shurson et al., 2021). These biosecurity restrictions also limit the capability of evaluating ASFV survival and inactivation in various feed ingredients under real world feed supply chain demonstrations because unlike many RNA viruses (Dee et al., 2018), no suitable surrogate has been available for ASFV.

African swine fever virus is a member of the *Asfarviridae* family which is part of a larger group of virus families that are classified as nucleocytoplasmic large DNA viruses (NCLDVs) and evolved from a common ancestor. These NCLDVs are found in a variety of environments, and can infect humans (*Poxviridae*), fish (*Iridoviridae*), insects (*Ascoviridae*), swine (*Asfarviridae*), amoeba (*Marseilleviridae* and *Mimiviridae*) and algae (*Phycodnaviridae*) (Iyer et al., 2001, 2006; Yutin et al., 2009; Colson et al., 2013). Until now, no surrogate NCLDV with similar features to that of ASFV, nor any other virus with suitable surrogate properties, have been proposed for use in studies to evaluate ASFV survival and inactivation in feed ingredients and complete feeds. *Emiliania huxleyi* virus strain 86 (EhV-86) is an ecologically important NCLDV which controls blooms of the marine unicellular phytoplankton *Emiliania huxleyi* (Schroeder et al., 2002; Allen et al., 2006) and shares many important features with ASFV (Balestreri et al., 2022). Both ASFV and EhV-86 share many physical characteristics, such as complex virion ultrastructure and sensitivity profile to time and temperature exposure (Balestreri et al., 2022). In fact, EhV-86 has recently been shown to be one of the most thermally stable viruses known, with temperatures up to 100°C damaging most of the virus particles yet leaving a subset of intact and potentially viable particles for future re-infections. Given the similarities shared between ASFV and EhV-86, Balestreri et al. (2022) proposed the use of EhV-86 as a surrogate for ASFV.

There are many challenges involving various analytical methods and data interpretation when determining virus inactivation kinetics or survival in various types of feed matrices (Shurson et al., 2021). A common method used to measure virus inactivation is quantitative PCR or qPCR (De León et al., 2013; Shurson et al., 2021). The qPCR method is useful for quantifying the amount of virus nucleic acids in a sample, but it fails to distinguish nucleic acids from viable versus non-viable viruses. Viability PCR (V-PCR) is a relatively quick, new technique used to evaluate infectivity of a virus with the use of viable dyes, such as ethidium monoazide (EMA) or propidium monoazide (PMA) prior to nucleic acid extraction and PCR or RT-PCR evaluation (Moreno et al., 2015). Balestreri et al. (2022) developed a viability qPCR (V-qPCR) version of the technique that can be used to quantify the viable EhV-86 NCLDV from the background damaged viruses.

Considering the challenges of determining ASFV survival in feed ingredients under commercial conditions, we chose to use EhV-86 as a suitable and safe surrogate for ASFV and employ the use of a new V-qPCR assay to quantify EhV-86 infectivity when recovered from selected feeds after an extended transport time period. This data can then be compared with the lab-based quantitative data on the half-life of ASFV Georgia 2007 in animal feed ingredients exposed to moderate temperature and humidity conditions simulating transoceanic shipment (Stoian et al., 2019). The main conclusion of this study was that longer virus half-lives in feed compared with half-lives in media support the concept that the feed matrix provides an environment that increases ASFV stability. We hypothesized that EhV-86 would survive in conventional and organic soybean meal and complete feed in real-world conditions similar to ASFV in simulated conditions. Specifically, we applied the V-qPCR method on three commonly used feed matrices inoculated with EhV-86 to determine whether this ASFV surrogate virus remains viable when exposed to the conditions of a 23-d transcontinental truck transport across the United States.

## Materials and methods

### Cell culture and EhV-86 stock

A culture of *Emiliania huxleyi* CCMP374 (courtesy of Dr. Martinez-Martinez laboratory, Bigelow – Laboratory for Ocean Sciences, East Boothbay, Maine) was grown in Alga-Gro® Seawater Medium (Carolina Biological Supply Company, Burlington, North Carolina) at 15°C with 18 h/6 h light/dark cycle (*ca.* 2400 lux) until the concentration of 2 × 10^5^ cells mL^−1^ was reached. Isolate EhV-86 (also courtesy of Dr. Martinez-Martinez laboratory) was added to *E. huxleyi* at a multiplicity of infection (MOI) of 1 and grown in a 15°C incubator until lysis was observed, which was usually after 4 d (Schroeder et al., 2002). The lysate was filtered through a 0.45 μm filter (Nalgene™ Rapid-Flow™ Bottle Top Filters, ThermoFisher Scientific, Waltham, Massachusetts) to remove cell debris. This filtration and infection procedure was repeated several times. The filtered lysate was divided into aliquots and kept in the dark at 4°C until use.

### Feed matrices

Previous research results have shown that swine viruses, such as ASFV, can survive in various experimentally-inoculated feed matrices including conventional and organic soybean meal (Dee et al., 2016, 2018). Therefore, feed ingredients used in this study included conventional solvent extracted, dehulled soybean meal (SBMC: containing 1–2% oil and 46–47% crude protein), organic mechanically extracted soybean meal (SBMO: containing 6–7% oil and 44–45% crude protein), and a complete grower-finisher swine feed (*CF*: corn and soybean meal-based). For each feed matrix, four subsamples (30 g each) were weighed and placed into individual 50 ml mini-bioreactor tubes with vented caps. A total of three allotments per feed matrix were spiked with 2 ml EhV-86 (1 × 10^9^ viruses mL^−1^ as calculated by qPCR and flow cytometry (Balestreri et al., 2022) *via* injection using a 3 ml syringe with an 18-gauge needle. The remaining three samples served as negative controls with no virus added to the feed matrix.

### Transport model

**Figure 1:**
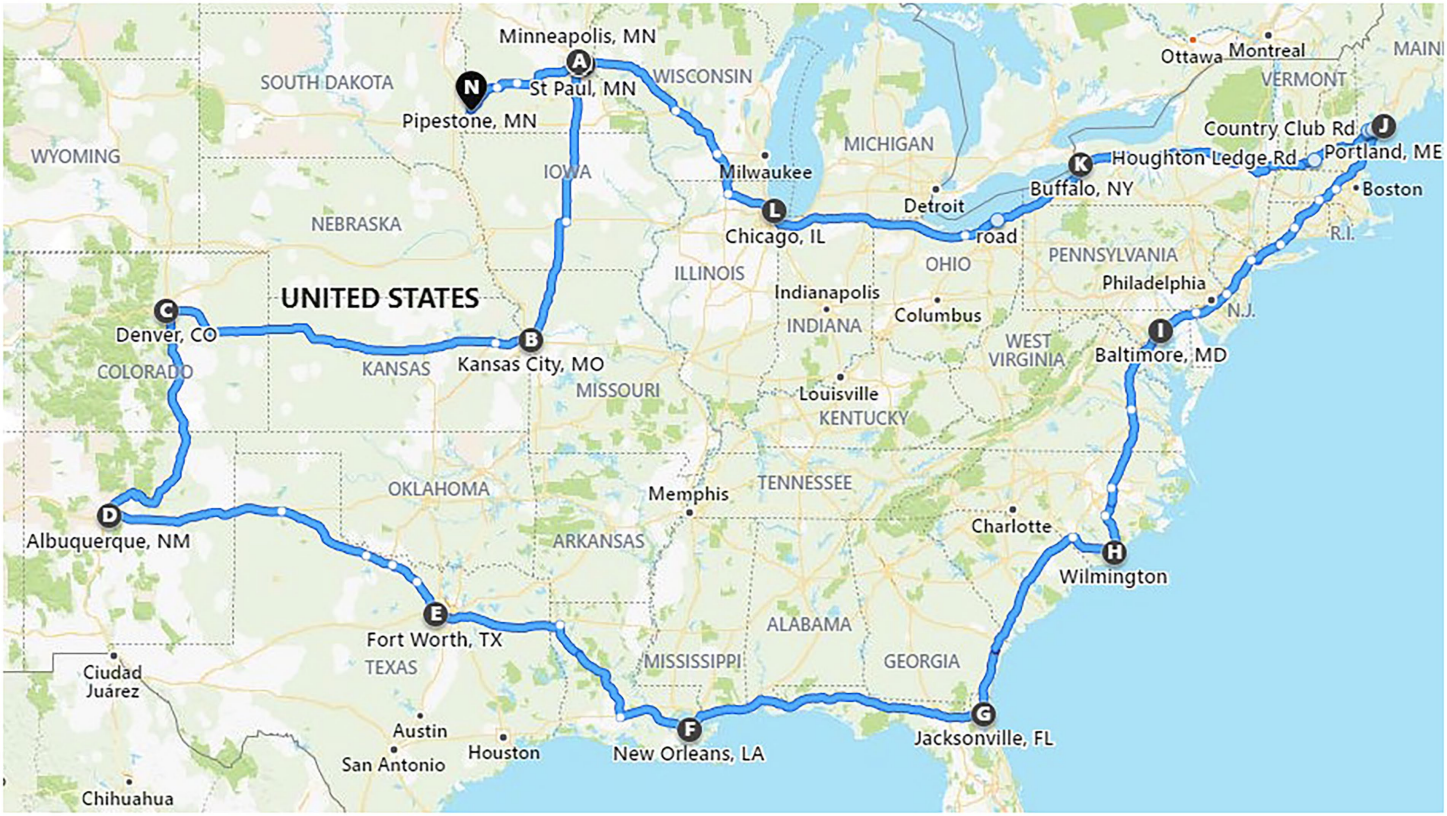
Map of the route traveled by a commercial truck carrying test feed ingredients inoculated with *Emiliania huxleyi* virus strain 86. Cities and towns indicated by letters of the alphabet show the journey start (A) and end points (N), with overnight stays labelled from B to M (adapted from Dee et al., 2021).

All feed samples were placed in a box on the trailer floor of a commercial semi-truck with a 15.8 m trailer (Csp Delivery, Fridley, Minnesota, USA). The truck carrying the inoculated feed samples departed from Minneapolis, Minnesota on November 30, 2020 and returned on December 22, 2020 (23 days). The route covered various regions of the United States including 29 states in the Midwest, Rocky Mountains, Southwest, Gulf Coast, Eastern Seaboard, New England region, and the Great Lakes region (Figure 1). The temperature and humidity were recorded every 15 min during transport and were reported by Dee et al. (2021). The commercial truck did not encounter any unexpected stops or accidents. The goal of this route was to cover several regions of the United States and expose the feed ingredients and viruses to a wide variety of environmental conditions. The temperature ranged for 1–17.5°C within the feed with the relative humidity ranging from 20 to 68% (Dee et al., 2021). Upon completion of the journey, samples were removed from the truck and stored at −20°C until analysis.

### Testing of protocol for EhV-86 elution from soybean meal

Two hundred μL of EhV-86 filtrate (1 × 10^9^ viruses mL^−1^) was added to 1 g of SBMC in a 50 ml Falcon tube (Corning™ Falcon 50 ml Conical Centrifuge Tubes, ThermoFisher Scientific, Waltham, Massachusetts), and held at room temperature for 5 min before eluting the virus from the soybean meal by adding 10 ml of Alga-Gro® Seawater Medium (Carolina Biological Supply Company, Burlington, North Carolina). The tube was vortexed repeatedly for 1 min before incubating in a water bath (Isotemp 205 Digital Water Bath, ThermoFisher Scientific, Waltham, Massachusetts) set at 40°C for a total time of 30 min. The tube was removed and vortexed every 5 min for 30 s. At the end of the temperature exposure, the tube was centrifuged at 4700 rpm for 5 min to collect the soybean meal at the bottom of the tube. The supernatant (virus eluant) was removed and filtered through 0.22 μm syringe filter (Millex™-GP Sterile Syringe Filters with PES Membrane, MilliporeSigma™, Waltham, Massachusetts) into an Amicon centrifugal tube (Millipore Amicon Ultra 15 ml, MilliporeSigma™, Waltham, Massachusetts). The virus eluant was washed 3 times with 1X phosphate buffered saline (1 × PBS, ThermoFisher Scientific, Waltham, Massachusetts) *via* centrifugation as per manufacturer’s instructions, and the final 200 μl volume obtained was split into two aliquots of 100 μl each. This process was repeated to create biologically independent replicate samples.

### Standard (S-qPCR) and viability (V-qPCR) assays

All assays were conducted in triplicate. To one set of 100 μl virus eluants previously mentioned, PMAxx dye (Biotium Inc., Fremont, California; 25 μM final concentration) was added according to methods optimized by Balestreri et al. (2022) and represented the viability qPCR (V-qPCR) treatments. An untreated duplicate set (i.e., no addition of PMAxx dye) of samples served as a control template for standard qPCR (S-qPCR). All samples were incubated in the dark at room temperature for 10 min on a rocker for optimal mixing. The treated V-qPCR samples were then exposed for 30 min to light using PMA-Lite device (Biotium Inc., Fremont, California) to cross-link PMAxx dye to the DNA (free or within broken viruses). The duplicate S-qPCR samples were kept in the dark at room temperature for the same length of time. All samples were then used for DNA extraction (QIAamp® MinElute® Virus Spin, Qiagen, Valencia, California). The final 30 μl elution volumes were stored at 4°C until qPCR analysis was conducted.

One μL from all the samples (virus eluants and DNA extractions) served as the DNA template in the subsequent 20 μl qPCR mix (QuantiNova SYBR Green PCR kit, Qiagen, Valencia, California): 10 μl Master Mix, 0.1 μl QN ROX Reference Dye, 1.4 μl reverse primer (GACCTTTAGGCCAGGGAG, 0.7 μM final concentration), 1.4 μl forward primer (TTCGCGCTCGAGTCGATC, 0.7 μM final concentration), and 6.1 μl molecular grade water. The primers amplify part of the single copy major capsid protein (MCP) gene of EhV as described by Schroeder et al. (2003). The qPCR analysis was conducted using a QuantStudio™ 3 Real Time PCR machine (ThermoFisher Scientific, Waltham, Massachusetts) run on the following qPCR conditions: 2 min at 95°C followed by 40 cycles of 5 s at 95°C and 10 s at 60°C.

Standards for the qPCR assays were created using EhV-86 as the template, with the MCP amplificon purity confirmed using E-Gel electrophoresis system (ThermoFisher Scientific, Waltham, Massachusetts) and extracted using Zymoclean™ Gel DNA Recovery Kit (Zymo Research, Irvine, California). The number of EhV-86 genomic copies that equate to MCP copies in our extracted MCP amplicon product was calculated using the following formula:


$$Noofcopies=\frac{\left(ng\times 6.022\times 10^{23}\right)}{83454.93Da\times 1\times 10^{9}}$$

where ng is the amount of the MCP amplicon as measured by Qubit4 (Invitrogen, ThermoFisher Scientific, Waltham, Massachusetts), 6.022 × 10^23^ is Avogadro’s number, 83454.93 Da is the molecular weight of our MCP amplicon as calculated using the Sequence Manipulation Suite (Stothard, 2000), and 1 × 10^9^ is used to convert the molecular weight of the amplicon to nanograms. A dilution series of the MCP amplicon was used to create a EhV-86 genomic equivalent standard curve. Fresh dilutions for the standard curve were made for every qPCR run.

### Protocol for EhV-86 elution from feed matrices used in transport study

One gram of each 30 g of feed matrix was sampled on d 23 and was used in the virus elution protocol as previously described. The qPCRs were carried out on DNA extracted from the eluant. The percentage of virus recovered was calculated as follows:


$$\frac{\begin{array}{l} \mathrm{Amount of virus}\;\mathrm{per}\;\mathrm{gram}\;\;\\ \mathrm{recovered after treatment and analysis} \end{array}}{\mathrm{Starting amount of virus}\;\mathrm{per}\;\mathrm{gram}\;}\times 100\%$$

### Statistical analysis

Visualization of data was performed using the ggplot2 package of RStudio environment (Version 1.1.456, RStudio, Inc., Boston, Massachusetts) using R programming language [Version 4.0.5 (2021-03-31), R Core Team, R Foundation for Statistical Computing, Vienna, Austria]. Viral quantity averages (virus μL^−1^) were normally distributed and compared using two-sample t-test assuming unequal variances in Excel.

## Results

### qPCR standards

**Figure 2:**
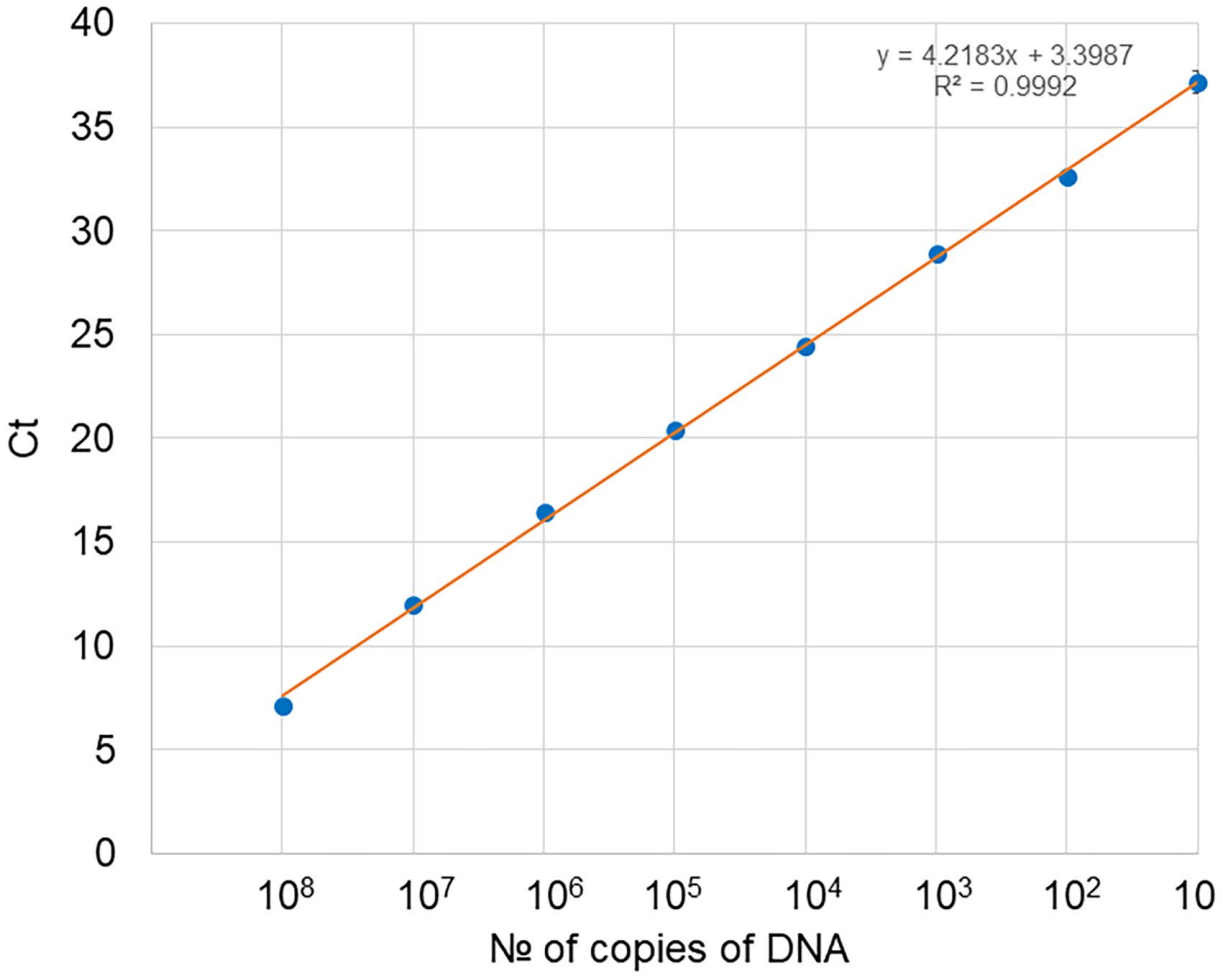
Standard curve obtained for EhV-86 PCR assays. Number of copies of DNA in the dilution series (1 μl used per dilution) against Ct (cycle threshold) values as obtained by real time PCR, with each dot representing three replicates. For 10^8^ copies of target DNA stock, the Ct was 7.16 and gradually increased to a final value of 37.16 with only 10 copies of DNA present.

A 10-fold serial dilution of the EhV-86 MCP qPCR amplicon was used to create a standard curve (*y* = 3.5926x + 7.0205) to convert the Ct values from the qPCR assay to EhV-86 genomic equivalents (Figure 2). The standard curve had an *R*^2^ value of 0.9996 indicating high accuracy of prediction (Bustin and Huggett, 2017). The EhV-86 MCP qPCR assay had a quantifiable range of 10–10 million EhV-86 genomes per reaction.

### Virus extraction efficiency of conventional soybean meal

**Figure 3:**
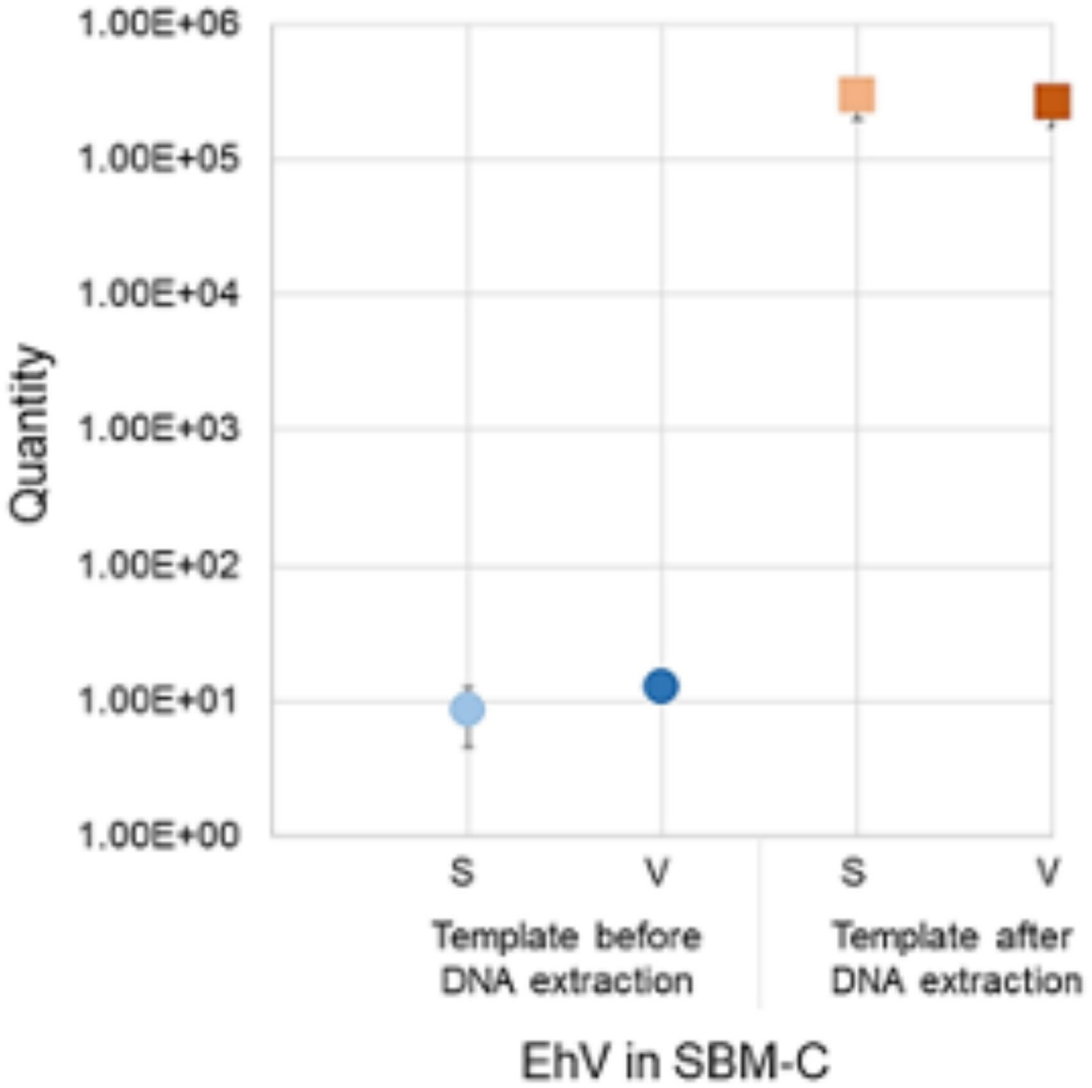
*Emiliania huxleyi* virus (EhV-86) quantity in conventional soybean meal (SBMC) as detected by S-qPCR and V-qPCR using EhV-86 eluant before (blue) and after extracting DNA (red). Bar = standard deviation from the mean.

Before performing multiple extractions from feed samples, we first evaluated the EhV-86 elution protocol using a SBMC sample. We also determined if the eluants could be used directly as templates in the qPCR or if EhV-86 DNA needed to be extracted from the final eluant. Concentrations of 8.53 × 10^0^ (± 4.0 SD, Ct 40.4) and 1.26 × 10^1^ (± 0 SD, Ct 38.2) EhV-86 μl^−1^ from the eluant in both standard (S) and viability (V) qPCRs, respectively, were obtained (Figure 3), which were equivalent to 4.26 × 10^2^ and 6.28 × 10^2^ EhV-86 g^−1^, or 0.01 and 0.01% recovery, respectively. In contrast, performing DNA extraction on the eluant significantly increased (*p* = 0.012) the amount of virus that was detected *via* standard (22.8%, ± 1.8 SD, Ct 19.2) and V-qPCR (19.9%; ± 1.6 SD, Ct 19.3) (Figure 3). Comparing the detection rates of both qPCR assays, we observed similar or identical recovery percentages (*p* = 0.81), suggesting that EhV-86 is stable in SBMC over the 5 min assay incubation period. Moreover, the elution efficiency of EhV-86 from SBMC was about 20% indicating that about 80% of the virus remained attached or associated to the feed matrix in some way.

### Virus DNA concentrations of feed matrices transported across the United States

**Figure 4:**
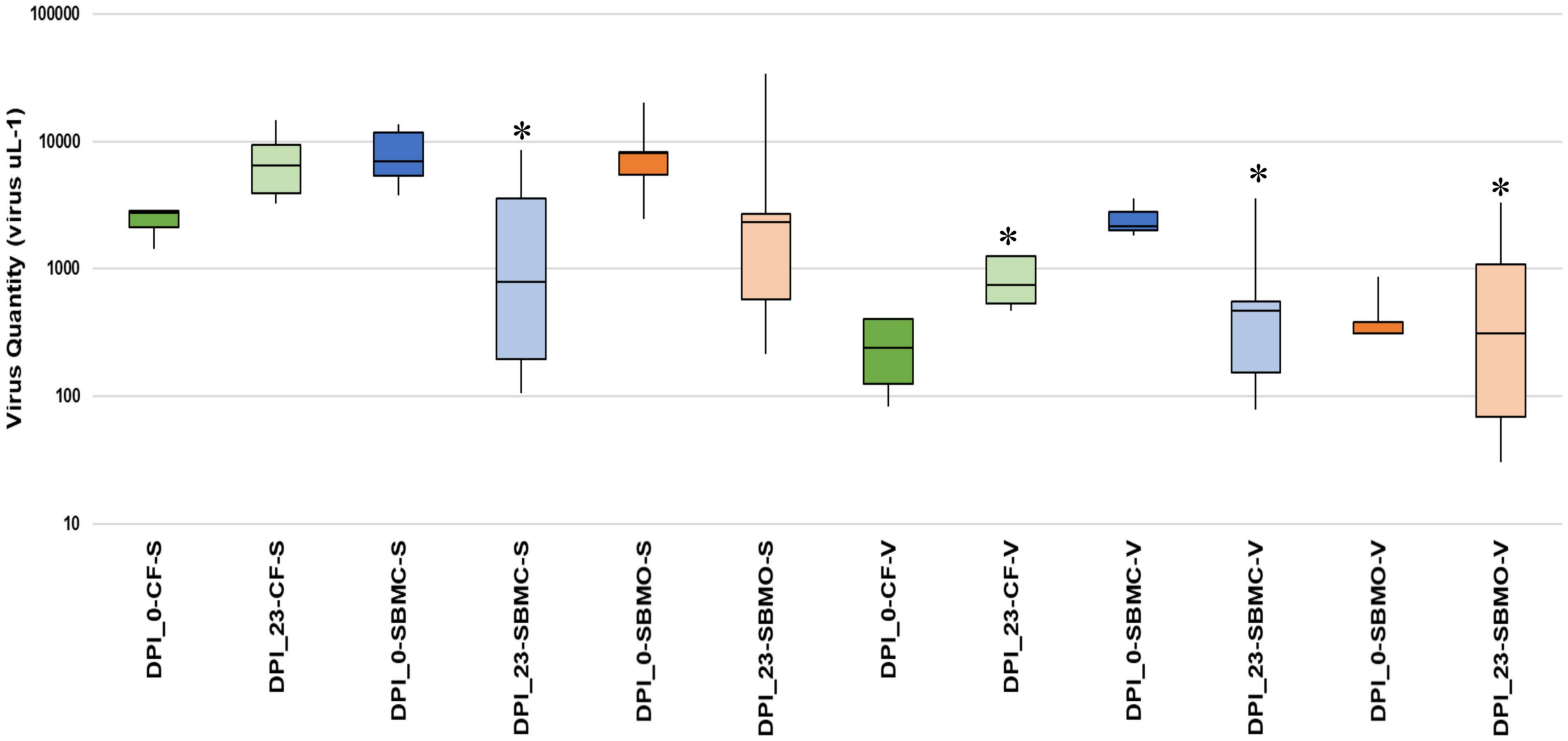
Boxplot showing the interquartile range of EhV-86 quantity (virus μL^−1^) in complete feed (*CF*_ green), conventional soybean meal (SBMC_blue), and organic soybean meal (SBMO_orange) with V-qPCR and S-qPCR. DNA presence on d 0 post infection (DPI_0) and after 23-d commercial truck journey (DPI_23). Asterisks represent the significant difference in average virus recovered between day 23 samples when comparing *CF*-S to SBMC-S, SBMC-V, SBMO-V and *CF*-V, *p* = 0.006, 0.001, 0.002 and 0.001, respectively, as determined by ANOVA (Tukey’s test) at 95% confidence level. The line extending from the top and the bottom of the boxes are the upper (maximum) and lower (minimum) limits, respectively. The middle line in the box in the median. The upper box is Q3, the upper quartile or 75th percentile. The lower box is Q1, the lower quartile or 25th percentile.

On d 0, 30 g of each type of feed sample was inoculated with 2 ml of EhV-86 at a concentration of 1 × 10^9^ viruses mL^−1^, which resulted in an initial virus load of 6.6 × 10^7^ viruses g^−1^ of feed matrix. For the inoculated *CF* samples, an average of 2.36 × 10^3^ EhV-86 μl^−1^ of eluant (Ct 24.9) or 1.18 × 10^5^ EhV-86 g^−1^ of *CF* was recovered on d 0, which represented only a 0.2% average recovery rate. In inoculated SBMC and SBMO samples, 6.83 × 10^3^ EhV-86 μl^−1^ (Ct 23.3) or 3.41 × 10^5^ EhV-86 g^−1^, and 8.61 × 10^3^ EhV-86 μl^−1^ (Ct 23.2) or 4.31 × 10^5^ EhV-86 g^−1^, respectively, were recovered. The average recovery rates for SBMC and SBMO were 0.52 and 0.65%, respectively (Figure 4). In addition, of the three feed matrices evaluated, the least variation of EhV-86 content from the mean was in *CF* (2.10 × 10^3^ and 2.82 × 10^3^ for the 1st and 3rd quartiles, respectively). There was also greater EhV-86 deviation from the mean in SBMC (5.39 × 10^3^ and 1.17 × 10^4^ for the 1st and 3rd quartiles, respectively) than in SBMO (5.48 × 10^3^ and 2.82 × 10^3^ for the 1st and 3rd quartiles, respectively; Figure 4). These results suggest that sampling sensitivity is greatest in *CF* followed by SBMO and lastly SBMC and should be considered when evaluating results to determine the most effective virus inactivation methods.

After the 23-days commercial trucking journey across the United States, an average of 6.81 × 10^3^ EhV-86 μl^−1^ (Ct 22.2) or 3.41 × 10^5^ EhV-86 g^−1^ was recovered from the *CF* matrix. This represents an average recovery rate of 0.52%, with a 289% increase in virus concentration compared to d 0 (Figure 4). Similarly, an average of 1.87 × 10^3^ EhV-86 μl^−1^ (Ct 25.3) or 9.37 × 10^4^ EhV-86 g^−1^ was detected in SBMC (0.14% recovery or 28% more viruses) on d 23. For SMBO, an average of 6.25 × 10^3^ EhV-86 μl^−1^ (Ct 24.0) or 3.13 × 10^5^ EhV-86 g^−1^ was recovered, which equates to 0.47% recovery and a 73% increase in virus concentration compared to EhV-86 concentrations on d 0 (Figure 4). As previously described, the deviation from the mean was greater in the SBM matrices (SMBC = 1.96 × 10^2^ and 2.14 × 10^3^ for the 1^st^ and 3^rd^ quartiles, respectively, and SBMO = 5.76 × 10^2^ and 6.40 × 10^3^ for the 1^st^ and 3^rd^ quartiles, respectively) compared to the *CF* (3.90 × 10^3^ and 9.40 × 10^3^ for the 1^st^ and 3^rd^ quartiles, respectively; Figure 4). These results indicate that there is greater variation of virus quantity recovered in the SBM matrices compared with *CF* which implies that sampling sensitivity is greatest in *CF* followed by the SBM matrices.

### Viable virus concentrations of feed matrices transported across the United States

On d 0, an average of 2.19 × 10^2^ EhV-86 μl^−1^ (Ct 29.0) or 1.10 × 10^4^ EhV-86 g^−1^ viable virus was detected with the V-qPCR assay from the *CF* matrix. These concentrations represent an average of 0.02% recovery rate and 9% viability at the start of the study when compared with the eluted virus counts on d 0. On d 23, an average of 7.46 × 10^2^ EhV-86 μl^−1^ (Ct 25.5) or 3.73 × 10^4^ EhV-86 g^−1^ was recovered representing an average recovery rate of 0.06%. This resulted in a range from 11 to 32% in virus viability depending on whether the d 23 or d 0 standard qPCR results were used as baselines, respectively. These results indicate that EhV-86 viability increased by 2 to 22%, and no loss in viability due to the 23-d transport event.

Similarly, an average of 2.08 × 10^3^ EhV-86 μl^−1^ (Ct 25.1) or 1.04 × 10^5^ EhV-86 g^−1^ viable virus was detected on d 0 from SBMC. This represents an average 0.16% recovery rate and 31% viability at the beginning of the study. On d 23, 9.10 × 10^2^ EhV-86 μl^−1^ (Ct 26.2) or 4.55 × 10^4^ EhV-86 g^−1^ was measured, which equates to 0.07% recovery rate and a range in viability from 13 to 49%, depending on whether the d 0 or d 23 standard qPCR results were used as baselines, respectively (Figure 4). These results indicate a range in virus viability from a 17% loss to a gain of 18% compared with standard qPCR results. Given the large deviation from the mean for the 1^st^ and 3^rd^ quartiles observed for both the standard and viability qPCR data (Figure 4), no significant loss of virus was also observed in SBMC matrix over the 23-days transport period. Therefore, sampling methods are extremely important when evaluating the accuracy of virus survival results.

Finally, an average of 4.52 × 10^2^ EhV-86 μl^−1^ (Ct 27.7) or 2.26 × 10^2^ EhV-86 g^−1^ viable virus was detected on d 0 from SBMO, which represents an average 0.003% recovery rate and 0.05% viability at the start of the study. On d 23, 8.31 × 10^2^ EhV-86 μl^−1^ (Ct 27.1) or 4.15 × 10^4^ EhV-86 g^−1^ concentrations were obtained, which equate to a 0.06% recovery rate and a 10 to 13% range in viability depending on whether the d 0 or d 23 standard qPCR results were used as baselines, respectively. These results indicate that there was a gain of 10 to 13% in virus viability compared to standard qPCR results. As for the SBMC matrix, the large deviation from the mean for 1^st^ and 3^rd^ quartiles observed for both the standard and viability qPCR data (Figure 4), indicates that no significant loss of virus occurred in the SBMO matrix over the 23-days transport period.

### Variation in EhV-86 concentrations among feed matrices

There was a large range in EhV-86 concentrations in the SBM matrices compared with the *CF* matrix (Figure 4). The average virus quantity for *CF* (standard qPCR) was significantly greater than in SBMC based on using both standard and viable qPCR methods, SBMO based on viable qPCR, and *CF* based on viable qPCR, with *p* = 0.006, 0.001, 0.002, and 0.001, respectively). The uninoculated negative control matrices had negative PCR results (no amplification observed after 40 cycles, data not shown) as expected.

### Assessment if PCR bias was associated with feed matrices

**Figure 5:**
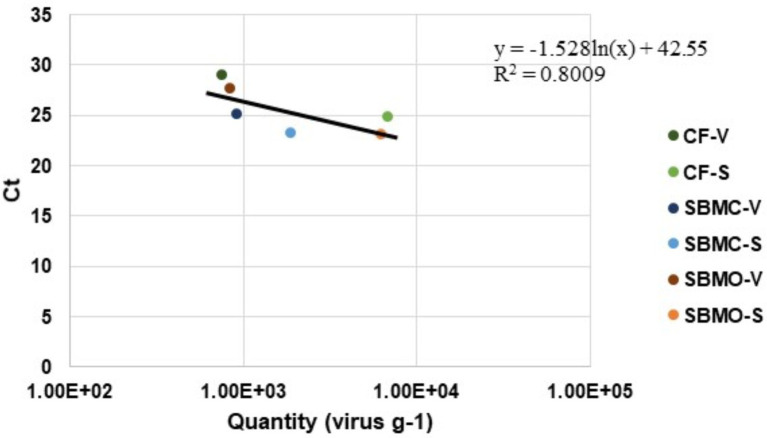
*Emiliania huxleyi* virus-86 quantity (virus g^−1^) relative to *C_t_* values in complete feed (CF), conventional soybean meal (SBMC), and organic soybean meal (SBMO) with viable (V) and non-viable or standard (S) DNA presence after a 23-d commercial truck journey.

A standard curve (*y* = −1.528ln(*x*) + 42.55) was created by plotting virus quantity (virus g^−1^) against Ct values in the various feed matrices using both viable or standard qPCR (Figure 5). The standard curve had an *R*^2^ value of 0.8009 which suggests that there were no inhibitors due to type of matrix in the qPCR assay, and was suitable for use in accurately quantifying amounts of virus for all of these feed matrices. The EhV-86 qPCR assay in feed matrices had a quantifiable range of 6,800 to 340,567 EhV-86 genomes per reaction (Figure 5).

## Discussion

In this study, the algal NCLDV EhV-86 was used as a surrogate for ASFV to study virus survival in selected feed matrices subjected to environmental conditions during a 23-days commercial trucking journey across the United States. Overall, viral load (virus μL^−1^) in samples was successfully quantified using the novel technology of the V-qPCR assay.

Current methods used to measure ASFV inactivation include hemadsorption (HAD_50_) tests, plaque assays, an EGFP-fluorescent technique, swine bioassays, median tissue culture infectious dose (TCID_50_) or cytopathic effect (CPE) assays, real-time RT-PCR and quantitative PCR (qPCR) (De León et al., 2013; Shurson et al., 2021). Although the quantification of the amount of virus nucleic acids using the qPCR method is useful, it fails to distinguish nucleic acids from viable versus non-viable viruses. Viability PCR utilizes viability dyes, such as ethidium monoazide (EMA) or propidium monoazide (PMA), prior to nucleic acid extraction and PCR or RT-PCR evaluation to evaluate infectivity of a virus (Moreno et al., 2015). Propidium monoazide (PMA) is a photoreactive, membrane-impermeant dye that will selectively penetrate cells which have compromised cell membranes, and thereby considered dead (Nocker et al., 2007; Elizaquível et al., 2014; Zhang et al., 2020). These dyes bind to the nucleic acids which then inhibits DNA from amplifying during PCR amplification (Nocker et al., 2007; Elizaquível et al., 2014; Zhang et al., 2020). Recently, viability RT-qPCR was used to evaluate the viability of PEDV exposed to heat treatments, with the goal of using this method to monitor PEDV contamination in feed and feed ingredients (Puente et al., 2022).

In this transcontinental United States transport scenario, EhV-86 viral DNA was present in the complete feed (average Ct value of 22.2 or 3.4 × 10^5^ virus g^−1^), conventional soybean meal (average Ct value of 25.3 or 9.37 × 10^4^ EhV-86 g^−1^), and organic soybean meal (average Ct value of 24.0 or 3.13 × 10^5^ EhV-86 g^−1^) after 23-days transport, which implies that NCLDVs like ASFV, are relatively stable in certain feed matrices (Dee et al., 2016, 2018, 2020b). In addition to detecting viral genome in these three types of feed matrices, viable EhV-86 was quantified in complete feed (average Ct value of 25.5 or 3.13 × 10^5^ EhV-86 g^−1^), conventional soybean meal (average Ct value of 26.2 or 4.55 × 10^4^ EhV-86 g^−1^), and organic soybean meal (average Ct value of 27.1 or 4.15 × 10^4^ EhV-86 g^−1^) after the 23-days transport period *via* viability qPCR. These results provide empirical evidence that the NCLDV ASFV-like *E. huxleyi* virus can remain viable in these three types of swine feed matrices for more than 3-wks. Among the three different feed matrices, the variation in the quantity of standard and viable DNA was greatest in the organic soybean meal and least in the complete feed. We also observed an increase in amount of virus detected in complete feed after the 23-days of transport time period compared with the amount on d 0 using the S-qPCR analysis, and an increase in the amount of viable virus detected in complete feed and organic soybean meal using the V-qPCR analysis. The factors contributing to these differences between feed matrices is unclear, but the likely reasons involve differences in their complexity and physiochemical properties (i.e., complex mixture of ingredients in complete feed compared with a single ingredient of soybean meal). Limited evidence suggests that moisture content and water activity of feed matrices may play a role in survival of some viruses in some feed matrices (Trudeau et al., 2016) but no studies have been conducted to evaluate this possibility with NCLDVs (Shurson et al., 2021). Understanding the amount and variation of viable virus in feed matrices is important because ASFV is extremely resilient and remains viable in a variety of environments and porcine tissues for many months (Mazur-Panasiuk et al., 2019). The minimum infectious dose of 10^4^ TCID_50_ that has been determined for ASFV demonstrates that ASFV can be transmitted orally through contaminated feed, and that repeated exposures exponentially increase the likelihood of infection (Niederwerder et al., 2019), which emphasizes the significance of our results regarding recovery of high concentrations of viable virus in these feed matrices under the environmental conditions of a 23-days transcontinental United States transport time period.

In their recent 23-days bulk transport study, Dee et al. (2021) found that RNA viruses (PEDV, SVA, and PRRSV) were present in one tonne totes of conventional soybean meal (average Ct values of 34.6, 35.7, and 34.9, respectively) and organic soybean meal (average Ct values of 37.6, 35.3 and 34.1, respectively) using real time RT-PCR. When evaluating totes with complete feed, SVA was recovered in only one tote (Ct value of 35.5), with no detectable PEDV or PRRSV found in inoculated totes using the same real time RT-PCR methodology. However, when pigs were fed contaminated complete feed, they developed a positive PEDV infection, but were negative for PRRSV, suggesting that PEDV remained infectious in a feed matrix after a trans-continental journey, while PRRSV did not (Dee et al., 2021). These observations are not surprising because of the lack of adequate sensitivity of commonly used diagnostic assays to characterize the quantity of viable virus capable of causing infection (Shurson et al., 2021). Furthermore, uneven virus distribution in bulk quantities of feed ingredients can easily occur due to localized contamination events, and depending on the sampling protocol used, could erroneously infer absence or presence of viruses when subsamples are taken. Although a sampling protocol has been developed for the detection of PEDV in soybean meal (Jones et al., 2019), it has not been validated for other viruses and feed ingredients (Shurson et al., 2021). Unfortunately, Dee et al. (2021) only reported mean Ct values for SVA, PEDV, and PRRSV which makes it impossible to determine if inadequate sampling or uneven virus distribution within the bulk feed mass contributed to this contradictory result. It must also be noted that the limitations with animal-based experiments and the consequent subsampling of animal products are overcome by using the V-qPCR method. Viable virus numbers can be accurately quantified down to 10 copies per PCR assay, before and after exposure using the identical methodology. This method is not reliant on whether symptoms are observed or whether the correct sample was collected from the animal at the correct time to confirm presence or absence of virus infection.

In our study, a minimum of 1,500 viable EhV-86 per gram of organic soybean meal was detected at the end of a 23-days transport period, which indicates that a pig consuming 1.4 kg of feed a day would be exposed to 9.26 × 10^5^ EhV-86 daily. If these values are representative for ASFV, then an ASFV infection would most likely be observed based on current estimates of the minimum infective dose (Niederwerder et al., 2019). However, it is important to note that these calculations are based on the methodological bias of EhV-86 being eluted from the feed. Results obtained in the current study showed that >80% of the NCDLV remained in the feed matrix, either in a potentially viable or degraded form, because we were only able to elute 20% of the added virus from the whole SBMC sample, which were all viable. Therefore, the remaining 80% of virus was not in the eluent. Only subsamples of infected feed (1 g from 30 g or 3.3% of the sample) could be analyzed at any one time. We observed mean virus recovery rates much less than 20%, with up to 99.9% of virus still retained in the solid matrix, indicating a potential subsampling effect, and thus detection sensitivity problem due to the non-homogenous distribution of virus across the sample. Consequently, accurate determination of the final exposure to the animal, herd, or farm based on subsampling data is difficult especially in low contamination scenarios. Further research is also required to address whether a similar virus retention rate is observed in other matrices and constituents used by the swine industry. With the help of the surrogate, *in situ* tests can be run in all real-world scenarios. Such experiments will provide invaluable information on the risk ASFV poses in current practices used in feed mills, refineries, processing plants and farm settings.

Currently, chemical mitigation strategies involving formaldehyde, medium chain fatty acids, and glycerol monolaurate have been shown to reduce ASFV infectivity in feed matrices in laboratory settings (Jackman et al., 2020; Niederwerder et al., 2020). These mitigation strategies need to be evaluated in full scale commercial feed mills and supply chains, which now appears to be possible by using a suitable surrogate for ASFV, such as EhV-86. The results of this study demonstrate the benefit of using a safe (non-infectious for animals, humans, or plants) ASFV-like NCLDV to make direct comparisons of ASFV survival and inactivation in various types of feed ingredients and complete feeds.

## Conclusion

Use of the NCLDV EhV-86 as a surrogate for ASFV in experimentally inoculated conventional and organic soybean meal and complete feed based on corn and soybean meal was present in a viable form after a 23-days transcontinental truck transport journey. However, sampling sensitivity rather than virus inactivation best explains the variation of in EhV-86 quantity detected in feed matrices after the 23-days transport period. These results demonstrate for the first time that ASFV-like NCLDVs can retain viability in swine feed matrices during long-term transport across the continental United States, thereby providing evidence for the use of EhV as a surrogate for ASFV for evaluating virus survival and inactivation under real-world demonstrations.

## Data availability statement

The original contributions presented in the study are included in the article/supplementary material, further inquiries can be directed to the corresponding authors.

## Author contributions

DS, Jv, PU, and GS designed the use of EhV-86 as a surrogate for ASFV. AP maintained the *Emiliania huxleyi* and EhV-86 stocks, performed the bioassays, ran the virus lysates on the flow cytometer, performed the viability qPCRs and wrote the manuscript. DS and HY designed the viability qPCR experiments. CB created the PCR EhV standards, optimized the viability qPCR, and prepared EhV-86 stock for the 23-day transport study. SD and AS designed and ran the 23-day transport study. DS supervised writing of the manuscript. All authors contributed to the article and approved the submitted version.

## Funding

This project was funded by SAM Nutrition.

## Conflict of interest

The authors declare that the research was conducted in the absence of any commercial or financial relationships that could be construed as a potential conflict of interest.

## Publisher’s note

All claims expressed in this article are solely those of the authors and do not necessarily represent those of their affiliated organizations, or those of the publisher, the editors and the reviewers. Any product that may be evaluated in this article, or claim that may be made by its manufacturer, is not guaranteed or endorsed by the publisher.

## Footnotes

^1^ https://empres-i.apps.fao.org/
